# Molecular association of pathogenetic contributors to pre-eclampsia (pre-eclampsia associome)

**DOI:** 10.1186/1752-0509-9-S2-S4

**Published:** 2015-04-15

**Authors:** Andrey S Glotov, Evgeny S Tiys, Elena S Vashukova, Vladimir S Pakin, Pavel S Demenkov, Olga V Saik, Timofey V Ivanisenko, Olga N Arzhanova, Elena V Mozgovaya, Marina S Zainulina, Nikolay A Kolchanov, Vladislav S Baranov, Vladimir A Ivanisenko

**Affiliations:** 1 Federal State Budget scientific Institution "The Research Institute of Obstetrics, Gynecology and Reproductology named after D.O. Ott", St. Petersburg, Russia; 2 Saint-Petersburg State University, St. Petersburg, Russia; 3 The Institute of Cytology and Genetics of the Siberian Branch of the Russian Academy of Sciences, Novosibirsk, Russia; 4 Novosibirsk State University, Novosibirsk, Russia

**Keywords:** automated text analysis, text-mining, ANDSystem, associative networks, disease associome, pre-eclampsia, diabetes mellitus, gestational diabetes, obesity

## Abstract

**Background:**

Pre-eclampsia is the most common complication occurring during pregnancy. In the majority of cases, it is concurrent with other pathologies in a comorbid manner (frequent co-occurrences in patients), such as diabetes mellitus, gestational diabetes and obesity. Providing bronchial asthma, pulmonary tuberculosis, certain neurodegenerative diseases and cancers as examples, we have shown previously that pairs of inversely comorbid pathologies (rare co-occurrences in patients) are more closely related to each other at the molecular genetic level compared with randomly generated pairs of diseases. Data in the literature concerning the causes of pre-eclampsia are abundant. However, the key mechanisms triggering this disease that are initiated by other pathological processes are thus far unknown. The aim of this work was to analyse the characteristic features of genetic networks that describe interactions between comorbid diseases, using pre-eclampsia as a case in point.

**Results:**

The use of ANDSystem, Pathway Studio and STRING computer tools based on text-mining and database-mining approaches allowed us to reconstruct associative networks, representing molecular genetic interactions between genes, associated concurrently with comorbid disease pairs, including pre-eclampsia, diabetes mellitus, gestational diabetes and obesity. It was found that these associative networks statistically differed in the number of genes and interactions between them from those built for randomly chosen pairs of diseases. The associative network connecting all four diseases was composed of 16 genes (*PLAT, ADIPOQ, ADRB3, LEPR, HP, TGFB1, TNFA, INS, CRP, CSRP1, IGFBP1, MBL2, ACE, ESR1, SHBG, ADA*). Such an analysis allowed us to reveal differential gene risk factors for these diseases, and to propose certain, most probable, theoretical mechanisms of pre-eclampsia development in pregnant women. The mechanisms may include the following pathways: [TGFB1 or TNFA]-[IL1B]-[pre-eclampsia]; [TNFA or INS]-[NOS3]-[pre-eclampsia]; [INS]-[HSPA4 or CLU]-[pre-eclampsia]; [ACE]-[MTHFR]-[pre-eclampsia].

**Conclusions:**

For pre-eclampsia, diabetes mellitus, gestational diabetes and obesity, we showed that the size and connectivity of the associative molecular genetic networks, which describe interactions between comorbid diseases, statistically exceeded the size and connectivity of those built for randomly chosen pairs of diseases. Recently, we have shown a similar result for inversely comorbid diseases. This suggests that comorbid and inversely comorbid diseases have common features concerning structural organization of associative molecular genetic networks.

## Background

Pre-eclampsia (PE) is the leading cause of maternal and foetal morbidity and mortality. It is a pregnancy complication, predominantly occurring after 20-weeks of gestation, as well as in labour, and it is characterized by multiple organ dysfunction syndromes, including the dysfunction of the kidneys, liver, vascular and nervous systems, and the foetoplacental complex [1,2]. The general clinical symptoms of PE are oedema, proteinuria and hypertension. The clinical outcome of PE may not always be predictable. Either form of PE can be extremely insidious, rapidly progressing, and, even in the absence of one of its general symptoms, may lead to life threatening complications for the mother and foetus [3]. In 70-80% of cases, PE is secondary to an underlying disease [1]. Pre-eclampsia risk factors include cardiovascular diseases (arterial hypertension), kidney, liver and gastrointestinal tract diseases, endocrine disorders (obesity, diabetes mellitus), and autoimmune diseases (antiphospholipid syndrome) [1,3,4]. According to meta-analysis data, women with a history of PE have 1.79 times the risk of venous thromboembolism, 1.81 times the risk of stroke, 2.16 times the risk of ischemic heart disease and 3.7 times the risk of hypertensive disease, compared with women without PE [5]. Thus far, it remains unclear whether the presence of pathological processes before pregnancy predisposes one to PE, or whether defects in multiple organs and systems, induced by PE, are responsible for the development of extragenital diseases in the future. Such joint manifestations of diseases are called comorbidities [6] or syntropies [7]. Likewise, inversely comorbid [8] or dystropic [9] diseases statistically rare co-occur in patients as compared with co-occurrence that can be expected by chance. Previously, for asthma, tuberculosis, certain cancers and neurodegenerative diseases, we have shown that inversely comorbid diseases are more closely related to each other at the molecular level in comparison with randomly chosen pairs of diseases [10].

In recent years, bioinformatics methods have been widely used for modelling different pathological processes, analysing the molecular mechanisms of their development, identifying possible markers, and systematizing available data. Ample evidence regarding the influence of genetics on comorbidities has accumulated in the literature. Computer-based, text-mining methods were developed for efficient extraction of knowledge from the scientific literature. At the present time, COREMINE and MeSHOPs, which analyse the co-occurrence of biomedical terms [11,12], and STRING [13] and the MedScan system, which are based on the parsing of natural language texts [14], are widely used.

We have developed the ANDSystem, which was designed for the automated extraction of knowledge from natural language texts regarding the properties of molecular biological objects and their interactions in living systems [15]. Using this system, we have reconstructed protein-protein networks for proteins that are associated with water-salt metabolism and sodium deposition processes in healthy volunteers [16], as well as protein-protein interaction networks for *Helicobacter pylori*, which are associated with the functional divergence of *H. pylori*, isolated from patients with early gastric cancer [17]. We have also reconstructed associative networks representing molecular genetic interactions between proteins, genes, metabolites and molecular processes associated with myopia and glaucoma [18], and with cardiovascular diseases [19].

In the current study, we used the ANDSystem for the reconstruction of associative networks (the preeclampsia associome) representing molecular genetic interactions between genes associated with PE, diabetes mellitus (DM), gestational diabetes (GD) and obesity (Ob). We conducted an analysis of these networks to reveal differential and common risk factors for these diseases.

Finding pathways common to the indicated multifactorial diseases would contribute to a better understanding of the characteristic features of pre-eclampsia pathogenesis, as well as to the development of new diagnostic, preventative and therapeutic methods.

## Results

### Pre-eclampsia: its association, via comorbid genes, with diabetes mellitus, obesity and gestational diabetes

**Figure 1:**
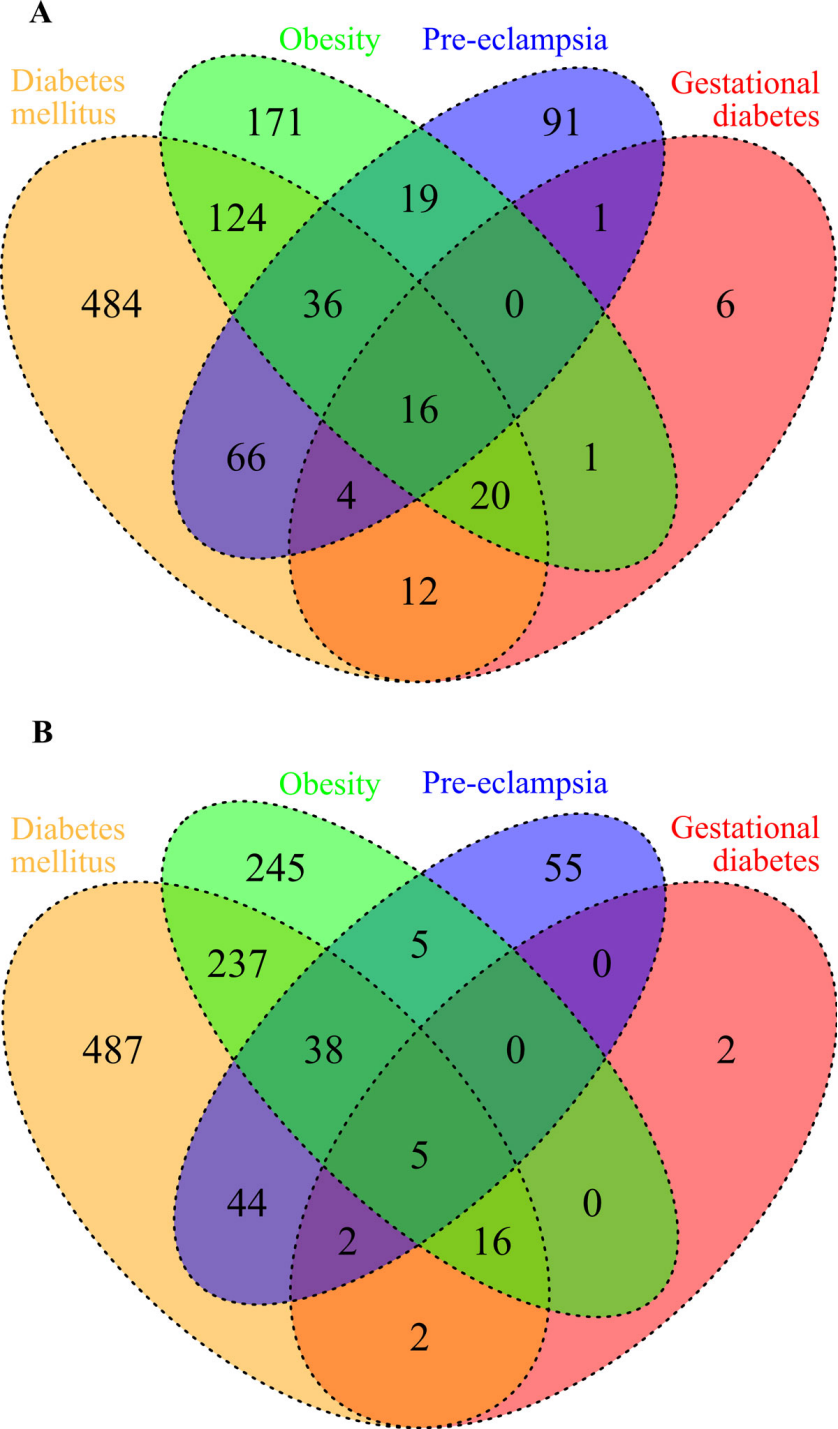
**Venn diagram demonstrating the intersections of the lists of genes associated with the analysed diseases (PE, DM, GD, Ob) according to the ANDSystem (A) and Pathway Studio (B)**.

The main goal of the current study was to identify comorbid genes whose dysfunction or mutation represent common risk factors for diseases that are concurrent with PE. To this end, we relied on published data [3,4] regarding the four most significant and widespread pathologies concurrent with PE: DM, Ob, GD and pyelonephritis. Furthermore, using the ANDSystem and Pathway Studio software, we reconstructed associative networks (disease-protein/gene-disease) comprising interactions between the above diseases via their associated genes. Subsequently, reduction was achieved by eliminating pyelonephritis, as genes associated with nephritis were not associated with PE and the other analysed disorders. Using the ANDSystem, we identified 1,051 proteins/genes associated with PE, Ob, DM and/or GD. Using Pathway Studio, 1,138 proteins/genes were identified. The results of both programs were in good agreement regarding the number of genes in groups associated with particular diseases (Figure 1). Unfortunately, we were not able to use STRING for the reconstruction of such networks, as this program does not provide information about protein/gene-disease associations.

**Figure 2:**
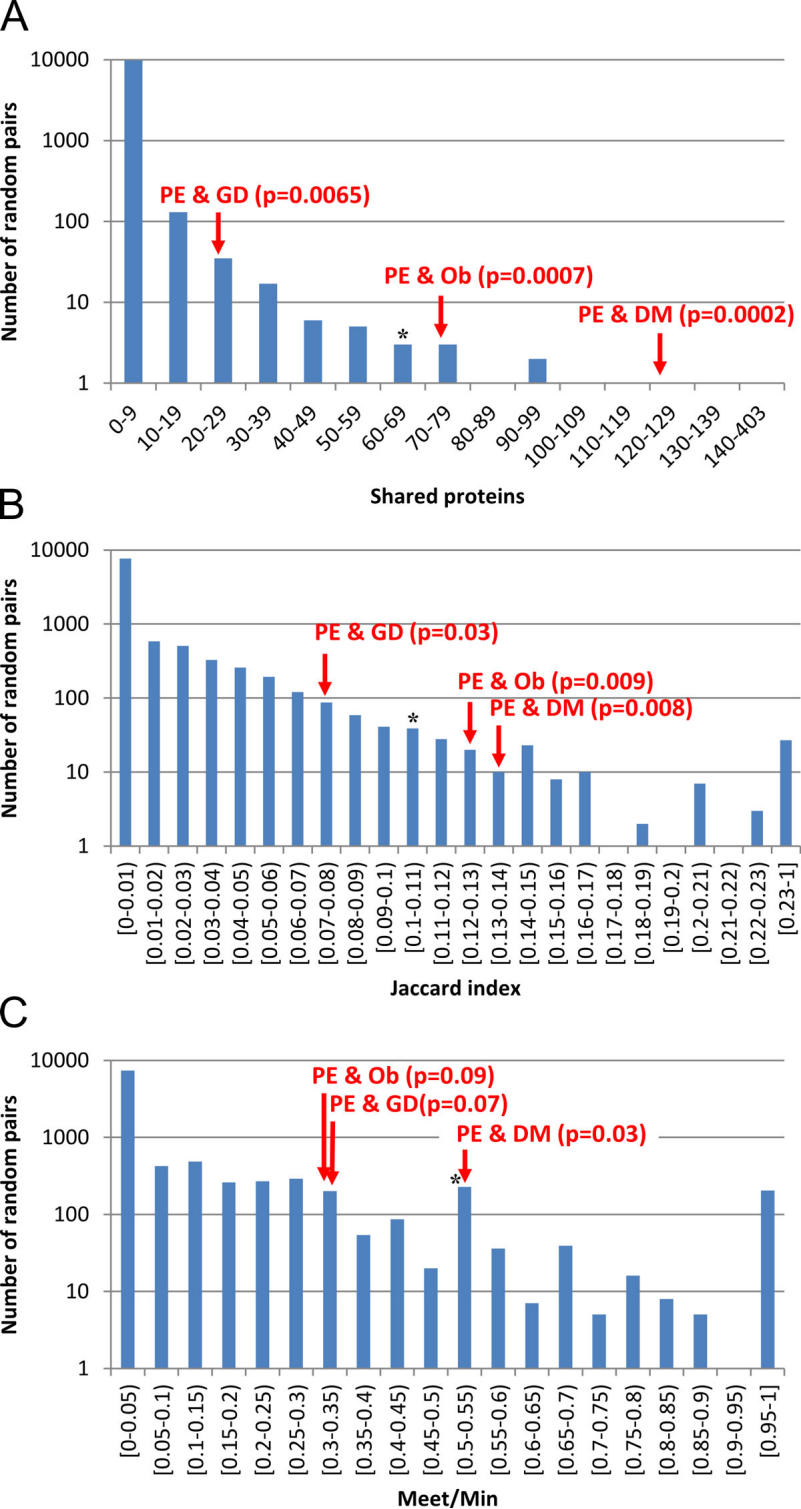
**Comparison of analysed and random networks by intersection (A), Jaccard (B) and Meet/Min (C) indices**. Bars show the distribution of the value for the features of the associative networks for randomly chosen disease pairs. Arrows indicate PE & GD, PE & DM and PE & Ob comorbid disease pairs. Asterisks indicate the position of inversely comorbid disease pairs (bronchial asthma and pulmonary tuberculosis) [10].

The number of proteins/genes common to different combinations of the examined diseases is shown in Figure 1. We assumed that comorbid diseases are more closely interrelated, via the common proteins/genes associated with them, as compared with randomly chosen disease pairs. To test this assumption, we calculated the distribution of three relation indices of random disease-protein/gene-disease networks built for random disease pairs: I_AB _(number of shared proteins), J_AB _(Jaccard index) and M_AB _(Meet/Min). All three disease pairs (PE & DM, PE & GD, PE & Ob) were significantly connected by the I_AB _and J_AB _indices at p < 0.05 (Figure 2). Only PE & DM pair was significantly different by M_AB _index (p < 0.05) from randomly generated pairs of diseases. Thus, PE and DM were found to be the most significantly associated disease pair for all three relation indices.

Next, we tested the hypothesis whether comorbid proteins/genes common to comorbid disease pairs interact more closely compared to a set of randomly chosen proteins/genes. Comparison of the associative molecular genetic networks with random ones demonstrated that the networks that describe the interactions between the comorbid proteins/genes for all three disease pairs (PE & GD, PE & DM, and PE & Ob) exhibited significantly greater connectivities than those of the random networks (p < 0.001).

**Figure 3:**
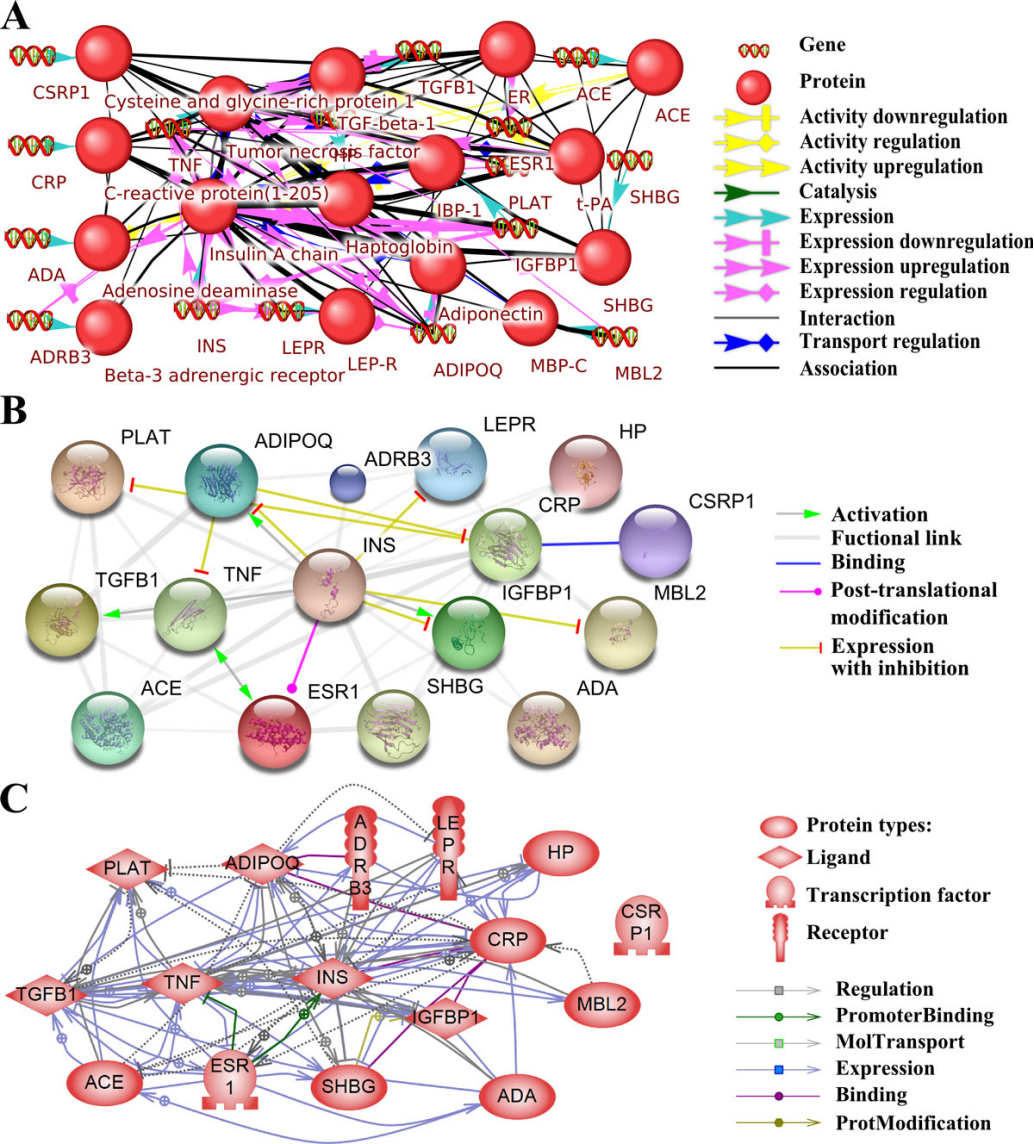
**ANDSystem (A), STRING (B) and Pathway Studio (C) networks describing shared proteins/genes**. Interactions between proteins/genes concurrently shared in PE, DM, Ob and GD are shown. In the ANDSystem network, proteins are shown as balls, and patterns with double helices designate genes. In the STRING network, proteins/genes are shown as balls (gene names are given). The colour and shape of the arrows indicate the type of association between the objects in all the networks.

Of particular interest was an appended analysis of the associative molecular genetic networks built for proteins associated concurrently with four comorbid diseases (PE, DM, Ob and GD). The three programs used to build this network were the ANDSystem, Pathway Studio and STRING (Figure 3). As Figure 3A shows, the ANDSystem network comprised 32 objects: 16 proteins and 16 genes, as well as 142 interactions. The ANDSystem has an advantageous feature: an object pair can also be associated concurrently with links of several types. For this reason, the number of associated object pairs, 87, was smaller than the number of links. The ANDSystem represented cases of the regulation of protein activity (six links), including up-regulation (two links) and down-regulation (three links) of protein activity; gene expression regulation (37 links), including up-regulation (seven links) and down-regulation (seven links); protein-protein interactions (two links); protein transport regulation (10 links); catalysis (one link); expression (16 links) and association (70 links). To compare the ANDSystem network with those of the STRING and Pathway Studio, the ANDSystem network was transformed into a protein-protein interaction network, with links from the genes assigned to their respective proteins, while links from genes as separate vertices were deleted from the network. Such a network contained 45 interconnected protein pairs.

The STRING network (Figure 3B) contained 16 proteins/genes, and 45 gene pairs connected by 47 links, including five different types: activation (four links), expression with inhibition (seven links), binding (one link), post-translational modification (one link), and functional links (34 links). The functional links in STRING were determined on the basis of Neighbourhood in the Genome, Gene Fusions, Co-occurrence Across Genomes, Co-Expression, Experimental/Biochemical Data, Association in Curated Databases, and Co-Mentioned in PubMed Abstracts [13].

The network built by Pathway Studio (Figure 3C) contained 16 proteins/genes, and 62 pairs of genes connected by 98 links, including six different types: binding (five links), expression (55 links), molecular transport (19 links), promoter binding (two links), protein modification (one link) and regulation (16 links).

There was a significant difference between the comorbid and random networks (p < 0.001), not only for disease pairs, but also for the associative molecular genetic networks that describe the interactions between proteins/genes associated concurrently with all four diseases (PE, DM, GD, Ob) (Figure 3A). These results demonstrated that comorbid proteins/genes are presumably involved in shared biological processes. This can explain the increased number of interactions between proteins/genes, as compared with those for associative molecular genetic networks of randomly chosen proteins/genes. Confirmation of this hypothesis would shed light on the molecular mechanisms underlying the interactions between comorbid diseases.

### Analysis of overrepresentation of Gene Ontology (GO) processes

Overrepresentation of GO biological processes was analysed for the group of proteins/genes associated with single diseases (PE, DM, GD and Ob) and pairs of diseases (PE & DM, PE & GD, PE & Ob), as well as concurrently with all four diseases. In each of these cases, more than 1,000 overrepresented processes were found (Additional file 1). Among these were a high number of quite general processes for which thousands of genes have been annotated. The connectivity rate (CR) was calculated for each process listed in Additional file 1 to check how closely the proteins/genes, which caused an overrepresentation of processes, interacted. After ranking the overrepresented biological processes according to the CRs, 313 processes had the highest CR (equal to 1) (see Additional file 1). Just as expected, generalized, nonspecific biological processes had smaller CR values in the majority of cases as compared with specialized processes involving a relatively small number of genes.

Among the overrepresented biological processes with a maximum CR were positive regulation of monooxygenase activity, regulation of fat cell differentiation, regulation of lipid metabolic process, nitric oxide and carbon monoxide metabolism, regulation of protein kinase B signalling cascade, regulation of NF-kappa B transcription factor activity, regulation of glucose metabolism and transport, regulation of cellular response to oxidative stress, regulation of cytokine production, regulation of cell cycle process and others. Thus, the use of the CR index in the GO enrichment analysis revealed the specific GO processes and lower the rank of less informative general processes.

### Reconstruction of associative pathways describing potential molecular mechanisms via comorbid genes involved in overrepresented GO biological processes

The next step of the current study was to reconstruct the molecular pathways connecting PE with DM, Ob, and GD, via interactions between the specific and comorbid genes. The Pathway Discovery module of the ANDVisio software was used to trace separate pathways in the network of molecular genetic interactions associated concurrently with all four pathologies. The Pathway Discovery module was used to search for pathways in the network using patterns set by the user.

**Figure 4:**
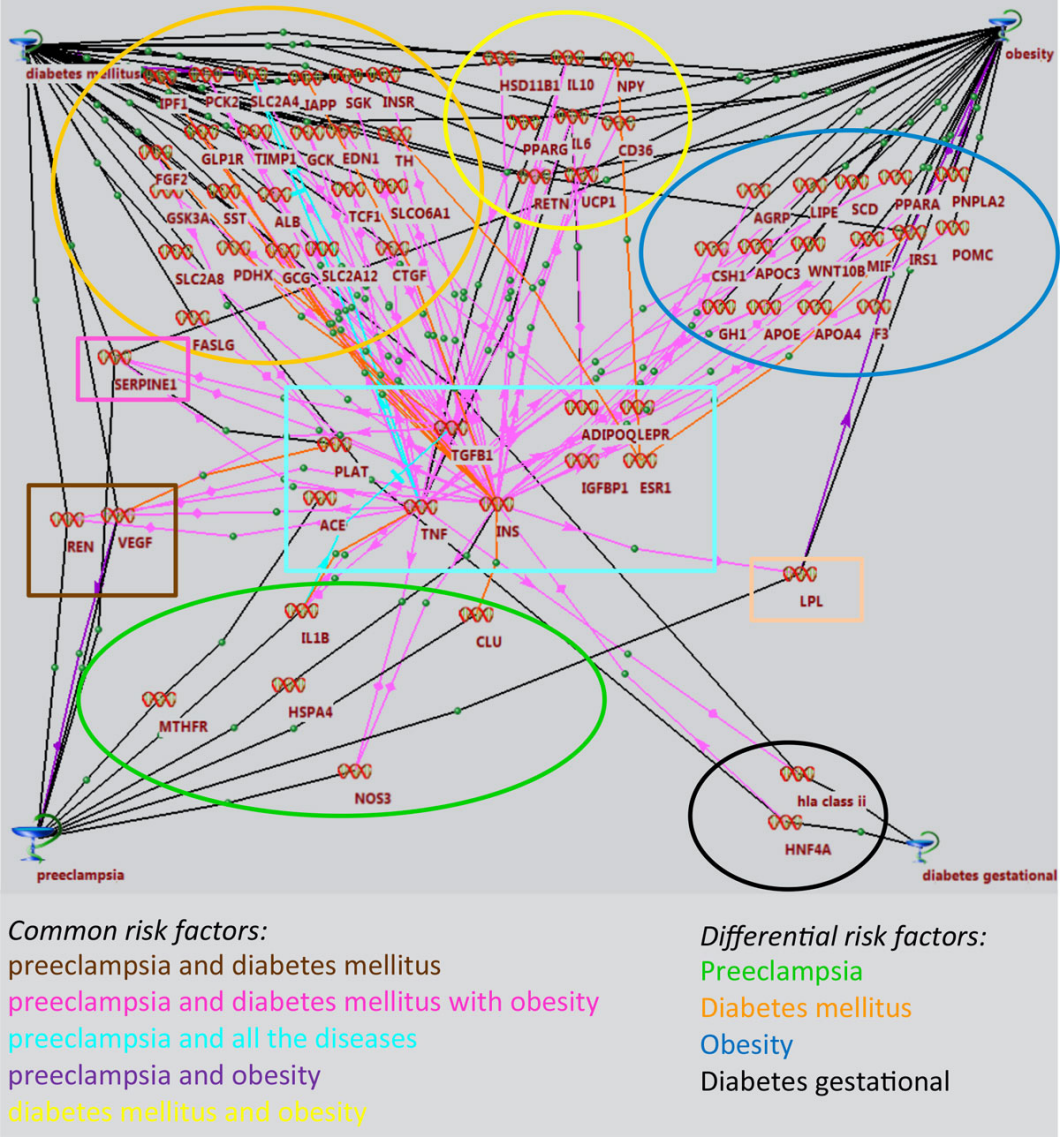
**Associative network comprising genes connecting PE with DM, OB and GD**.

The patterns were of the following type: <PE> - <any protein/gene specific to PE> - <any comorbid protein/gene> - <any protein/gene specific to Ob or GD, or DM> - <Ob or GD, or DM>. The program chose all the pathways meeting the pattern's criteria: the starting link was PE; the second link of the chain should be one of the proteins/genes associated with PE, exceptions were proteins/genes comorbid for all four diseases (4-comorbid); the third link should be one of the 4-comorbid proteins/genes (PLAT, ADIPOQ, ADRB3, LEPR, HP, TGFB1, TNFA, INS, CRP, CSRP1, IGFBP1, MBL2, ACE, ESR1, SHBG, ADA); the fourth link should be one of the proteins specific to Ob, GD, or DM, with the exception of 4-comorbid proteins/genes. The last link should be one of the diseases (Ob, GD or DM). The total number of identified pathways was more than 50. These were combined into a single pathway network (Figure 4).

Common, as well as specific, risk factors were distinguished for the following combinations of diseases: PE and DM; PE and Ob; PE and DM, Ob; PE and DM, Ob, GD; (see Figure 4). The largest number of connections was obtained for the *TNFA, TGFB1 *and *INS *genes, which revealed specialized GO processes with maximum CRs, such as: «positive regulation of protein kinase B signalling», «cascade regulation of NF-kappa B transcription factor activity», «regulation of mitosis», «regulation of nuclear division», «regulation of protein secretion MAPK cascade», «positive regulation of protein transport», «regulation of protein complex assembly», «positive regulation of cell migration», «positive regulation of secretion», «positive regulation of cellular component movement», «positive regulation of organelle organization», «regulation of mitotic cell cycle», «regulation of immune effector process», «intracellular protein kinase cascade», «regulation of cellular component biogenesis», «regulation of cell cycle process», «regulation of organelle organization», «regulation of cell cycle» (see Additional file 1).

An associative pathway network connecting PE, via the *PLAT, ADIPOQ, LEPR, TGFB1, TNFA, INS, IGFBP1, ACE *and *ESR1 *genes, with DM, OB and GD incorporated 66 genes with 167 connections (see Figure 4). Most of these connections (78) corresponded to the "association" type (shown in black). Sixty-nine of them could be referred to "expression regulation" types and 13 as "co-expression" (shown in red); eight comprised "down regulation", "degradation regulation", and "degradation downregulation" (shown in violet).

The differential network of PE risk factors included seven genes (interleukin-1-beta (IL1B), endothelial (NOS3) NO-synthase, heat shock 70 kDa protein 4 (HSPA4), apolipoprotein J (CLU) and 5,10-methylenetetrahydrofolate reductase (MTHFR).

Thus, whereas all the identified PE risk factors might be treated as potential markers of this disease, the most probable molecular mechanism underlying PE, DM, OB and GD includes the pathway starting from the *TGFB1, TNFA, INS *and *ACE *genes, through the *IL1B, NOS3, HSPA4 *(HSP74), *CLU *and *MTHFR *genes, and eventually to PE.

Thus, the probable chains of molecular events on the way to combined PE, in this context, are as follows: TGFB1 or TNFA - IL1B - PE; TNFA or INS - NOS3 - PE; INS - HSPA4 or CLU - PE; ACE - MTHFR - PE.

## Discussion

The associative networks analysed in this work (see Figures 1, 2, 3, 4) appeared to be significant for the understanding of the nature of PE, thereby supporting the hypothesis that PE represents a stable complex of clinical manifestations [1,3,4]. The key players in the reconstructed networks are comorbid genes which, on the one hand, contribute to the development of PE and its pathogenically related disorders, and, on the other hand, may play the role of "triggers" in the presence of other pre-eclampsia-promoting factors (genes and proteins). Comorbid genes are characteristic of many multifactorial diseases [20]. Moreover, many comorbid diseases may involve various pathophysiological mechanisms [20], and the construction of associative networks makes it possible to understand their molecular interrelations.

An analysis of reconstructed associative networks, which describe interactions between comorbid proteins/genes associated with different pair combinations of PE with DM, Ob, and GD, demonstrated that comorbid diseases differ in a statistically significant manner from random disease pairs. The differences concern both the number of common genes associated with the diseases and the interactions between such genes. The number of vertices in the comorbid networks, as well as the number of interactions between the vertices, exceeded those of random disease pairs. At the same time, the density of connections in the associative molecular genetic network describing the interactions between proteins/genes associated concurrently with all four diseases also differed significantly from those of the random networks formed by random sets of proteins/genes. Interestingly, we also observed the same regularity for inversely comorbid diseases [10]. It has been shown that the associative networks reconstructed for pairs of inversely comorbid diseases, including bronchial asthma and pulmonary tuberculosis, as well as nine pairs formed by neurodegenerative (Parkinson disease, schizophrenia, Alzheimer disease) and cancer diseases (colorectal neoplasms, prostatic neoplasms, lung neoplasms), significantly differed from the networks that describe interactions between random diseases. An example of the mutual arrangement of inversely comorbid (bronchial asthma and pulmonary tuberculosis) and comorbid diseases is shown in Figure 2.

Our current results are in many respects consistent with those of epidemiological studies worldwide. It has been amply demonstrated that the common risk factors of PE were DM, Ob and GD [1,2,21-26]. In most studies, DM is a leading risk factor, as it occurs in more than half of the women with PE [1,2,24]. Furthermore, DM is more strongly associated with a late-onset of the disease, which prevails among all the cases [24,25]. A study of twin gestations supports our reasoning. In this study, an evaluation of associated factors in PE gestations and a comparison of the incidence of pregnancy complications among twins with and without PE demonstrated that a high pregnancy body mass index (BMI) and diabetes were associated with PE [27].

We identified 16 genes encoding shared proteins in the molecular network, built using the literature- and database-mining (ANDSystem, Pathway Studio and STRING), that simultaneously connected with PE, DM, GD and Ob. Most shared genes determined in this study encode proteins controlling energy metabolism, and are associated with the immune response and inflammation.

An analysis of the associations of these genes with PE and DM, GD and Ob obtained in case-control, family-based, and meta-analyses studies, which we conducted using the HuGE Navigator, revealed that 14 of the 16 shared genes were associated with at least one of the diseases (see Table 1). Two genes (*CSRP1 *and *PLAT*) had never been shown to be associated with PE and DM, GD and Ob. Four shared genes (*ACE, ADIPOQ, MBL2, TNFA) *were found to be associated with all the diseases.

**Table 1:** Statistics of gene-disease associations for PE, DM, GD and Ob obtained with the HuGE Navigator.

Gene name	PE	DM	GD	Ob
*ACE*	39	244	2	77
*ADA*	-	6	-	-
*ADIPOQ*	4	156	4	176
*ADRB3*	1	49	4	145
*CRP*	2	20	-	28
*CSRP1 *	-	-	-	-
*ESR1*	7	21	-	36
*HP*	2	36	-	5
*IGFBP1*	-	7	-	5
*INS*	1	88	4	26
*LEPR*	7	35	1	154
*MBL2*	4	14	1	1
*PLAT*	2	3	-	1
*SHBG *	-	6	1	7
*TGFB1*	8	33	-	8
*TNFA*	24	132	5	83

The number of associations determined by case-control, family-based and meta-analysis studies are shown.

We believe that the identification of these genes in the current study is of importance because they encode proteins important for the development of diseases, as confirmed by experimental studies (Table 1).

Angiotensin-converting enzyme (ACE) plays a key role in regulating blood pressure by influencing vascular tone by activating the vasoconstrictor angiotensin II and inactivating the vasodilatory peptide bradykinin. Inter-individual differences in blood ACE levels are at least in part explained by the presence of an insertion/deletion (I/D) polymorphism in intron 16 of the ACE gene, with higher ACE levels observed in D allele carriers. The results of many studies confirmed the association of ACE polymorphism with PE [28]. Other studies have indicated that the ACE gene is a factor that contributes to the manifestation of GD [29], diabetic nephropathy and Ob [30,31].

It has been shown that polymorphisms in the adiponectin gene (*ADIPOQ*) modulate the circulating concentration of adiponectin. Abnormal adiponectin levels, as well as *ADIPOQ *polymorphisms, have been associated with PE [32]. Some variants of this gene are associated with the occurrence of GD [33], while other polymorphisms may contribute to type 2 DM risk [34] and Ob in adults [35].

Mannose-binding lectin (MBL) is involved in the maintenance of an inflammatory environment in the uterus. High MBL levels have been associated with successful pregnancies, whereas low levels are involved in PE development. Association between polymorphisms in the structural and promoter regions of the *MBL2 *gene and PE have been evaluated [36]. MBL gene polymorphisms are associated with GD and with type 2 DM [37,38]; in addition, MBL deficiency may confer a risk of Ob and insulin resistance [39].

Tumour necrosis factor-alpha (TNF-α) participates in the immune response and inflammation. Many studies have showed that there is an association between the *TNFA *gene and PE among Europeans [2,40]. The -308 G-->A polymorphism of the *TNFA *promoter gene is involved in the pathophysiology of insulin resistance and GD [41]. The same polymorphism is a genetic risk factor for the development of type 2 DM [42]. Individuals who carry the -308A *TNFA *gene variant have a 23% greater risk of developing obesity compared with controls, and they showed significantly higher systolic arterial blood pressure and plasma insulin levels, supporting the hypothesis that the *TNFA *gene is involved in the pathogenesis of the metabolic syndrome [43].

The PE associome contains more links than each of the individual networks. The identified, shared genes have been classified according to GO. Such a network was needed for a GO overrepresentation analysis. The presence of processes identified by the GO analysis in the pathogenesis of PE is not surprising. The central hypothesis of our understanding of PE is that it results from ischaemia of the placenta, which in turn releases factors into the maternal circulation that are capable of inducing the clinical manifestations of the disease [2]. Multiple pathogenetic mechanisms have been implicated in this disorder, including an imbalance between angiogenic and anti-angiogenic factors, autoantibodies to the type-1 angiotensin II receptor, platelet and thrombin activation, defective deep placentation, intravascular inflammation, endothelial cell activation and/or dysfunction, and oxidative and endoplasmic reticulum stress that promote the differentiation of trophoblasts from a proliferative to an invasive phenotype, regulate cell homeostasis through their involvement in post-translational modifications and protein folding, and induce the release of proinflammatory cytokines and chemokines. Other mechanisms include hypoxia and trophoblast invasion, which down-regulate the expression of transforming growth factor β3 (TGF-β3) and hypoxia-inducible factors (HIF-1α and HIF-2α) [2,44]. These results indicated the contribution of common, non-specific, pathological processes to the development of PE, DG, GD and Ob.

In addition to the identification of common proteins/genes associated with different pathological processes, another goal of the study was to find unique markers for PE. To do so, we reconstructed potential mechanisms of molecular interactions using the ANDSystem software, a program that allows the identification of the largest number of links (see Figure 4). Although the central network core of these pathways contained only nine common genes (*PLAT, ADIPOQ, LEPR, TGFB1, TNFA, INS, IGFBP1, ACE, ESR1*), it incorporated 68 genes with 174 connections between them, and differential factor risks of PE were identified: the *IL1B, NOS3, HSPA4, CLU *and *MTHFR *genes. The contributions of many of these genes to the pathogenesis of PE has been confirmed by numerous studies [2,45-50]. Here, we showed for the first time that these genes can be specifically involved in the pathogenesis of PE. However, it is not yet clear why these genes have a greater involvement in PE. The possible trigger mechanisms of combined PE are linked to the processes that are carried out by the products of the identified genes, namely, inflammation (*IL1B*), endothelial dysfunction (*NOS3*), heat shock and stress (*HSPA4*), stabilizing cell membranes at diverse fluid-tissue interfaces and protecting the vascular endothelium from an attack by some factors in plasma, such as active complement complexes (*CLU*), and homocysteine metabolism (*MTHFR*).

In addition, the results are of particular importance in regard to the theory of confounding assumptions as false mechanisms of genetic association when the factor is associated with a confound, but not the phenotype, and a confound, in turn, is associated with the phenotype [51,52]. The identified genes can act as such a confound.

## Conclusions

The current results broaden our knowledge of the molecular mechanisms of the interactions between comorbid diseases. This reconstruction of associative molecular genetic networks that describe interactions between PE and comorbid diseases (GD, Ob, and DM) differed significantly from partner networks built for random disease pairs. Networks between PE and comorbid diseases had a larger number of genes and links between them. With this in mind, it is of interest that similar features of associative network structure have been observed for inversely comorbid diseases [10]. It can be suggested that comorbid and inversely comorbid relationships between diseases involve larger sets of closely interrelated genes larger than those for random pairs of diseases. In the future, we intend to perform a scale analysis that connects different disease pairs to detect potential comorbid/inversely comorbid diseases for all the possible disease pairs via which these diseases can interact. Reconstruction and analysis of the PE associome is useful for revealing the genetic factors involved in the pathogenesis of the disease and for identifying its differential risk factors, as well as for modelling the theoretical mechanisms of PE development in pregnant women with underlying diseases, such as DB, Ob or GD.

## Methods

We used three systems that allowed the automated reconstruction of networks that describe the interactions between proteins/genes and diseases: STRING [13], Pathway Studio [14] and ANDSystem [15].

The ANDSystem was developed for the automated extraction of facts and knowledge regarding the relationships between proteins, genes, metabolites, microRNAs, cellular components, molecular processes, and their associations with diseases from published scientific texts and databases. To extract knowledge from texts in the ANDSystem, the shallow parsing method was applied. Pathway Studio is a software application developed for the navigation and analysis of biological pathways, gene regulation networks and protein interaction maps. The program uses the natural language processing approach to extract knowledge from the texts of scientific publications. STRING is a database and a web resource that contains information about protein-protein interactions (including physical and functional interactions) that is mainly based on the use of text-mining methods.

The associative networks for the considered disease pairs were graphs whose vertices were diseases and human proteins/genes, while the edges were the associations between diseases and proteins.

The following indices of relation between a pair of associative networks were used: (1) the intersection index, $I_{AB}=\left|A\cap B\right|$ equal to the intersection size of protein sets A and B composed of proteins concurrently associated with diseases D_A _and D_B_; (2) the Jaccard index [53] was calculated as the ratio of $I_{AB}$ to the combination of sets A and B involving at least one of the diseases D_A _and D_B_, $J_{AB}=\frac{I_{AB}}{\left|A\cup B\right|}$; (3) Meet/Min [54] was calculated as $M_{AB}=\frac{I_{AB}}{\text{min}\left(\left|A\right|,\left|B\right|\right)}$, where the denominator denotes the size of the minimum of sets A and B.

The statistical significance of the relation indices for the analysed diseases in the associative networks was determined by comparing these networks with the associative ones formed by pairs of randomly chosen diseases. For such an analysis, we used the ANDSystem because this program allows the comparison of reconstructed networks with random ones generated using the ANDCell knowledge base. All the interactions between proteins, genes, metabolites, diseases and other objects described by the ANDSystem are deposited in the ANDCell knowledge base, which is a module of this system [15]. The total number of diseases described in ANDCell was 4,075; of these, 991 were not found to be associated with any human protein. Such diseases were discarded from the analysis. To build the distribution of the relation indices for random disease pairs, 10,000 random disease pairs were generated (see Additional file 2). The P-value for the analysed disease pairs was calculated as the proportion of 10,000 random networks with the same or larger CR as in the examined pairs of diseases. The associative networks were reconstructed using the ANDSystem and Pathway Studio programs. STRING was not used for this purpose because it gave no information regarding interactions between protein/gene and diseases. The associative networks for the analysed disease pairs included only interactions of the disease-protein/gene type; the interactions between proteins/genes were discounted. As a result, to analyse the interactions between proteins/genes in the associative networks, additional protein/gene-protein/gene associative molecular genetic networks were built using the ANDSystem, Pathway Studio and STRING. The statistical significance of the connectivity of the associative molecular genetics networks built for the analysed disease pairs was also determined by comparing them with random networks. In such a case, for each analysed associative molecular genetic networks, 1,000 random networks were generated using the ANDSystem (only human proteins/genes were considered).

The statistical significance (p-value) of the difference between the connectivity of the analysed network and that of the random networks was also determined, like in the case of the associative networks, as the proportion of random networks with the same or greater number of links between the vertices compared with the number of links in the analysed network. The random molecular genetic networks were built according to the following rules. Proteins/genes considered as vertices in the random networks were taken from the ANDCell knowledge base. To ensure that the proteins/genes in the random networks were represented at a level of study close to that of the proteins/genes from the analysed networks, we considered only those random proteins/genes whose connectivity rate was the same as connectivity rate of proteins/genes from the analysed networks. The set *Q_i _*was formed for each *i*-th vertex of the analysed network. *Q_i _*was composed of all the proteins/genes from the ANDCell knowledge base having an interaction number in ANDCell equal to the protein/gene interactions in the knowledge base represented by the *i*-th vertex. The protein/gene for the *i*-th vertex of the random network was chosen by chance for the set *Q_i _*. The links between the vertices in the random networks were set according to the interactions described in the ANDCell knowledge base.

The results of the automated extraction of information regarding the interactions between proteins/genes and diseases were tested manually. The recognition correctness of the object names in the text, as well as the presence of their interactions, was tested. The lists of shared and specific proteins were reduced by expert evaluation to retain only those participating in the pathogenesis of both diseases for shared proteins, and in the pathogenesis of either disease for specific proteins, as shown previously [10].

The BINGO tool [55] was used to evaluate the overrepresentation of the biological processes for the considered protein/gene set. The enrichment was evaluated using a hypergeometric test with the Benjamini and Hochberg FDR correction using the whole annotation as a reference set. The human Uniprot-GOA Gene Association file (release 2013_05) was used as the custom annotation file. In addition to the statistical significance of the overrepresentation, the overrepresented GO processes were characterized by the CR of the respective proteins/genes in the associative molecular genetics network built for intersection of the four studied diseases. The CR for the protein group of the examined network involved in the overrepresented GO biological process was calculated as the ratio of the number of the protein pairs connected by the network protein pairs of the given group to the number of all possible pairwise combinations of proteins of this group. As is known, the reconstruction quality of the molecular genetic networks is related frequently to the problem of the completeness of information regarding the interactions between proteins. For this reason, to build the network, we took advantage of three independent programs: ANDSystem, Pathway Studio and STRING, with their parameters set by default.

## Competing interests

The authors declare that they have no competing interests.

## Authors' contributions

Expert analysis of the pathogenetic contributors (diabetes mellitus, gestational diabetes and obesity) was done by ASG, ESV, VSP, ONA, EVM, MSZ and VSB. The development of methods, programs, calculations and analyses of the structural organization of the molecular genetic networks was done by EST, PSD, OVS, TVI, NAK and VAI. All authors read and approved the final manuscript.

## Supplementary Material

Additional file 1 **Excel spreadsheet file containing information regarding the characteristics of overrepresented biological processes**. Click here for file

Additional file 2 **Excel spreadsheet file containing information regarding the distribution of the relation indices of the disease-protein-disease associative networks**. Click here for file

## Declarations

Publication of this article has been funded by the Russian Science Foundation grant No. 14-24-00123.

This article has been published as part of *BMC Systems Biology *Volume 9 Supplement 2, 2015: Selected articles from the IX International Conference on the Bioinformatics of Genome Regulation and Structure\Systems Biology (BGRS\SB-2014): Systems Biology. The full contents of the supplement are available online at http://www.biomedcentral.com/bmcsystbiol/supplements/9/S2.
